# Pegylated interferon-alfa-2a monotherapy in patients infected with HCV genotype 2 and importance of rapid virological response

**DOI:** 10.1186/1756-0500-4-316

**Published:** 2011-08-31

**Authors:** Reiko Etoh, Fumio Imazeki, Tomoko Kurihara, Kenichi Fukai, Keiichi Fujiwara, Makoto Arai, Tatsuo Kanda, Rintaro Mikata, Yutaka Yonemitsu, Osamu Yokosuka

**Affiliations:** 1 Department of Medicine and Clinical Oncology, Graduate School of Medicine, Chiba University, Chiba, Japan

## Abstract

**Background:**

Pegylated (PEG)-interferon (IFN)-alfa-2a plus ribavirin (RBV) therapy for 24 weeks is now a standard treatment protocol for patients with hepatitis C virus (HCV) genotype 2. As RBV cannot be used in certain situations, we examined whether PEG-IFN-alfa-2a monotherapy for 24 weeks or less would be sufficient to obtain a sustained virological response (SVR) in patients infected with HCV genotype 2.

**Methods:**

Forty-nine consecutive patients with HCV genotype 2 received PEG-IFN-alfa-2a (180 μg/week) subcutaneously without oral RBV for 8-64 weeks. HCV RNA level was determined by COBAS AMPLICOR HCV Test, v2.0.

**Results:**

HCV RNA was equal to or less than 100 KIU/mL (defined as low viral load) in 15 of 49 patients, and the remaining 34 had HCV RNA above 100 KIU/mL (defined as high viral load). All 15 patients with low viral load achieved rapid virological response (RVR; HCV RNA negative at week 4), and also achieved SVR with an average treatment duration of 17.1 weeks. The 34 patients with high viral load were treated for 33.7 weeks on average, and 19 of them (55.9%) achieved RVR. The SVR rates of these patients were significantly higher in those with RVR than without RVR (16/19 vs. 6/15 p = 0.0074).

**Conclusion:**

PEG-IFN-alfa-2a monotherapy for 24 weeks or less might be sufficient to treat selected patients with HCV genotype 2, especially those with low viral load and becoming negative for HCV RNA by week 4 of treatment.

## Background

Hepatitis C virus (HCV) is a major causative agent of chronic hepatitis, hepatic cirrhosis and hepatocellular carcinoma [1]. The number of cases of end-stage liver diseases caused by chronic HCV infection and being referred for liver transplantation is increasing rapidly [2]. The current standard care for chronic hepatitis C is based on the combination of pegylated-interferon (PEG-IFN) and ribavirin (RBV) for 48 weeks, and this treatment results in ~50% sustained virological response (SVR) in patients with HCV genotype 1 and high viral loads [3]. Among HCV genotypes other than HCV genotype 1, HCV genotypes 2/3 patients in particular show 70-80% SVR by this treatment for 24 weeks [4-6].

Recent studies revealed that a shorter course of therapy, for 12 weeks, with PEG-IFN-alfa-2b and RBV is as effective as a 24-week course for patients with certain HCV genotype 2/3 who show a rapid virological response (RVR) to treatment at 4 weeks [6], although treatment with PEG-IFN and RBV for 12 or 16 weeks in patients infected with HCV genotype 2 or 3 results in a lower overall SVR rate than treatment with the standard 24-week regimen [5,7].

On the other hand, PEG-IFN-alfa-2a without RBV induces SVR in some transplant recipients with recurrent hepatitis C [8] or in some dialysis patients infected with HCV [9]. In patients with cirrhosis, IFN, either alone or in combination with ribavirin, has been used cautiously, largely because it may exacerbate existing neutropenia and thrombocytopenia [10]. In general, PEG-IFN-alfa-2a monotherapy was better tolerated than the combination with RBV, although PEG-IFN-alfa-2a monotherapy was associated with a ~20% rate of SVR in patients infected with HCV genotype 1 [11].

Among pretreatment virological variables, the presence of HCV genotype 2 or 3 infection is the most powerful predictor of SVR [12], as patients with HCV genotype 2 are more likely to achieve SVR than those with genotype 1. Further, patients with lower titers of HCV RNA are also more likely to achieve SVR [13]. In this study, we sought to determine whether PEG-IFN monotherapy for fewer than 24 weeks would be effective in patients with HCV genotype 2.

## Methods

### Patients

In this study, we analyzed 49 patients infected with HCV genotype 2 at Chiba University Hospital between January 2004 and December 2008. All patients fulfilled the following criteria: 1) a history of abnormal liver function for more than 24 weeks, 2) positivity for anti-HCV antibody and HCV RNA, 3) HCV genotype 2, 4) negativity for HBs antigen, 5) negativity for anti-HIV, 6) no high titers of anti-nuclear antibodies, 7) no history of alcohol abuse, 8) not taking immunosuppressive drugs, and 9) liver biopsy findings compatible with chronic viral hepatitis.

Patients received PEG-IFN-alfa-2a (180 μg/week) subcutaneously without oral RBV for 8-64 weeks. The possible risks and benefits of shorter or longer treatment with PEG-IFN were explained to the patients so as to decide on a shorter (equal to or less than 24 weeks) or longer (more than 24 weeks) treatment protocol. This study was approved by the ethics committee of Chiba University, Japan (permission number G19033), and conformed to the Helsinki Declaration. Informed consent was obtained from all patients before enrollment in this study.

### Determination of HCV RNA titer and HCV genotype

Serum HCV RNA titer was measured using an Amplicor HCV monitor assay, version 2.0 (range: 0.5-850 KIU/mL) (Roche, Tokyo, Japan) [14]. HCV RNA was also examined qualitatively with the Amplicor HCV assay (Roche). The detection limit of this qualitative assay is 50 IU/mL, corresponding to 1.7 Log IU/mL by COBAS TaqMan PCR assay [15]. HCV genotype was determined using the antibody serotyping method of Tsukiyama-Kohara et al. [16,17]. In this serotyping assay, HCV serotypes 1 and 2 correspond to genotypes 1a/1b and 2a/2b, respectively, according to the classification of Simmonds et al. [18]. It was reported that in 84% patients, genotypes determined by this serological genotyping assay showed complete agreement with those determined by group-specific PCR, with none revealing a group opposite to that of the HCV genotype, and also that the detection rate of the serological genotyping assay was even higher than that of the PCR assay [17]. HCV RNA titer and HCV genotype were determined within 3 months prior to starting IFN treatment, and HCV RNA was assayed qualitatively at 24 weeks after the end of treatment.

### Serum liver function tests and hematology tests

Serum aminotransferase concentrations, other liver function tests and hematology tests were performed according to standard methods every 1 to 3 months before treatment, during treatment, and for at least 24 weeks after the end of treatment.

### Histological examination

Liver biopsies were performed within 6 months prior to the start of treatment in cases whose informed consent was obtained. Staging of fibrosis and grading of activity were based on the classification of Desmet et al. [19].

### Assessment of efficacy

The endpoint of the study was achievement of SVR, defined as seronegativity of HCV RNA throughout 24 weeks of post-treatment follow-up. RVR was defined as seronegativity of HCV RNA at 4 weeks of therapy. Patients who had undetectable HCV RNA within the initial 12 weeks of treatment were considered to have had complete early virological response (cEVR). End-of-treatment virological response (EOTVR) was defined as seronegativity of HCV RNA at the end of treatment. Relapse was defined as HCV RNA reappearance during the follow-up period in patients who had achieved an EOTVR [20].

### Statistical analysis

Statistical analysis was carried out using Student's t-test or chi-square test, and p < 0.05 was considered statistically significant.

## Results

### Profile of the patients

Forty-nine patients infected with HCV genotype 2 were enrolled. The HCV RNA level was 100 KIU/mL or more (defined as high viral load) in 34 and less than 100 KIU/mL (defined as low viral load) in 15 patients. Age, gender, ALT levels and platelet counts are shown in Table 1. As for histological findings [19], there were no significant differences between the high and low viral load groups (N = 7 and N = 21, respectively). Fibrosis staging of the high and low viral load groups was 1.5 ± 1.3 and 1.7 ± 0.9 (*P *= 0.65), and activity grading was 1.3 ± 0.5 and 1.8 ± 0.6 (*P *= 0.058), respectively. Of 49 patients enrolled, 43 were treatment-naïve, 5 had a history of IFN monotherapy and one was unknown. In the 15 patients with low viral load, only one patient had a history of IFN-alfa treatment. There were statistically significant differences of RVR between low viral load and high viral load groups (100% and 55.9%, respectively), and the two-tailed *P*-value was 0.0059 by chi-square test with Yates correction (Table 1).

**Table 1:** Demographic data of patients according to HCV RNA levels

Groups	Low Viral Load	High Viral Load	*P-*value
N	15	34	
Age (years)	50.8 ± 14.5	53.3 ± 11.7	*N.S*.
Gender (male/female)	11/4	21/13	*N.S*.
ALT (IU/L)	71.5 ± 56.5	100 ± 76.3	*N.S*.
Platelets (× 10^4^/mm^3^)	17.4 ± 5.5	100 ± 76.3	*N.S*.
HCV RNA (KIU/mL)	37.5 ± 26.3	459 ± 330	< 0.0001
Duration of treatment (days)	120 ± 80	236 ± 115	*N.S*.
RVR/non-RVR	15/0	19/15	0.0059

Low and high viral load were defined as < 100 KIU/mL and > 100 KIU/mL, respectively. ALT, alanine aminotransferase; RVR, rapid virological response; *N.S., statistically not significant*. Data are shown as mean ± S.D. Statistical analysis was carried out using Student's t-test or chi-square test. *P *< 0.05 was considered statistically significant.

### Treatment response in HCV genotype 2-patients with low viral load

**Figure 1:**
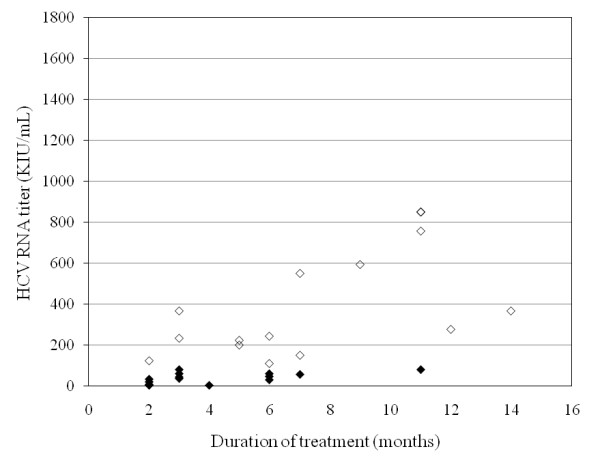
**Relationships between HCV viral loads and treatment duration in 31 patients with RVR and SVR**. 15 patients with low viral load and 16 patients with high viral load are shown as black and white diamonds, respectively.

The 15 patients with low viral load were treated for 17.1 ± 11.4 weeks (range, 7-44 weeks), and all achieved RVR as well as cEVR, EOTVR and SVR. None of them stopped treatment because of side effects (Figure 1). Of the 15 patients, 14 patients were treatment-naïve. The other patient, who had undergone IFN monotherapy, was retreated for 44 weeks and achieved SVR.

### Treatment response in HCV genotype 2-patients with high viral load

**Figure 2:**
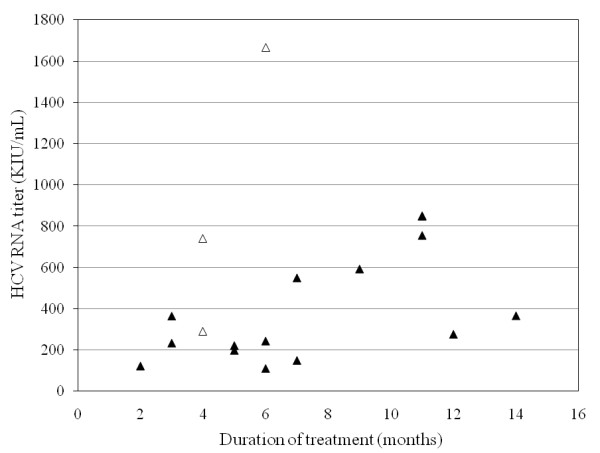
**Efficacy of IFN treatment in 19 patients with high viral load who were HCV RNA seronegative at week 4 according to HCV RNA load and duration of treatment**. Three cases had 850 KIU/mL and treated for 11 months. Black triangles, sustained virological response (SVR); white triangles, non-SVR.

**Figure 3:**
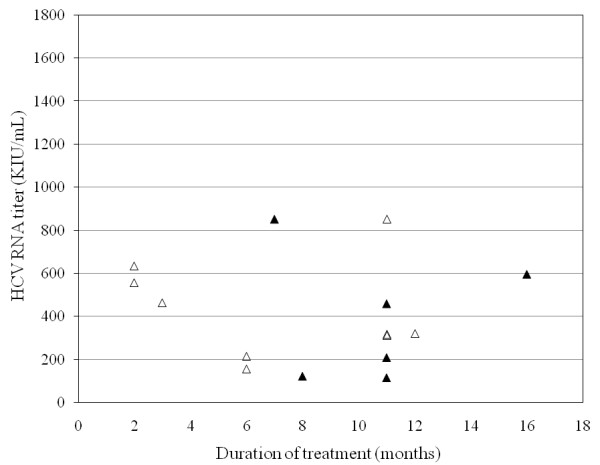
**Efficacy of IFN treatment of 15 patients with high viral load who were HCV RNA seropositive at week 4 according to HCV RNA load and duration of treatment**. Black triangles, sustained virological response (SVR); white triangles, non-SVR.

The 34 patients with high viral load were treated for 33.7 ± 16.4 weeks (range; 7-64 weeks). 22 (64.7%) had SVR, 4 (11.7%) relapsed and 4 (11.7%) did not respond. Five of them discontinued treatment at week 8, 11, 17, 22 and 25, respectively, due to abdominal pain, skin eruption and lack of virological response, and all but one failed to achieve SVR. RVR was achieved in 19 patients (55.9%), and SVR rates were significantly higher in those achieving RVR than in those who did not (16/19, 84.2%; 6/15, 40%, respectively; *P *= 0.0074) (Figures 2, 3). cEVR as well as EOTVR were achieved in 24 patients (70.6%), and SVR rates were significantly higher in those achieving cEVR than in those who did not (22/24, 91.7%; 0/10, 0%, respectively; *P *< 0.001).

### Treatment response in HCV genotype 2-patients with high viral load and RVR

Of the 19 patients with high viral load and RVR, SVR rates were 7/10 (70%) and 9/9 (100%) in patients treated for ≤24 weeks and > 24 weeks, respectively (*P *= 0.073). Of the 19 patients, 17 were treatment-naïve. The other 2 patients had been treated with IFN monotherapy, and one of them achieved SVR.

### Treatment response in HCV genotype 2-patients with high viral load without RVR

**Figure 4:**
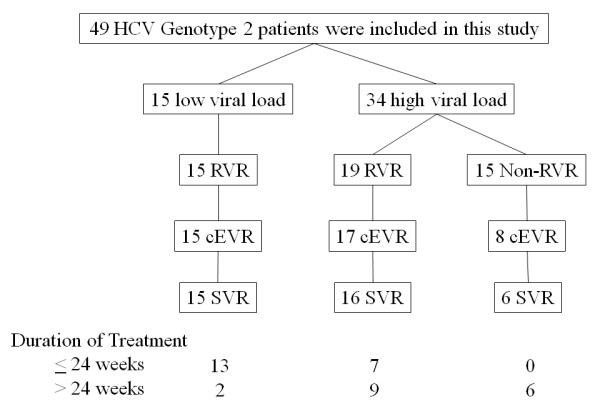
**Summary of this study**.

Of the 15 patients with high viral load and without RVR, SVR rates were 0/5 (0%) in patients treated for ≤24 weeks and 6/10 (60%) in those treated for > 24 weeks (*P *= 0.025) (Figure 4). Of the 15 patients, 12 patients were treatment-naïve. Two of the other patients had been treated with IFN monotherapy (one was unknown), and these 2 patients achieved SVR.

## Discussion

The present study showed that Japanese patients with HCV genotype 2 and undetectable HCV-RNA at week 4 of treatment achieved a high SVR rate with PEG-IFN monotherapy for 24 weeks or less. In Japan, between December 2003 and March 2007, until the national health insurance system approved the combination therapy PEG-IFN-alfa-2a with RBV, PEG-IFN-alfa-2a monotherapy was used at 180 μg once a week for 24-48 weeks for the treatment of chronic hepatitis C. Even now, we cannot use the combination therapy for HCV genotype 2 treatment-naïve patients with low viral loads under the national health insurance system in our country. Then, we used 180 μg of PEG-IFN-alfa-2a monotherapies for chronic hepatitis C patients and retrospectively analyzed the data during this period in the present study. This study was not a controlled trial, and various durations of treatment were adopted for the respective patients. However, the SVR rate of 100% in patients with low viral load and 17.1 weeks as average treatment duration and that of 84.2% in those with high viral load and undetectable HCV-RNA at week 4 of average treatment duration of 24 weeks were very high. Therefore, 24 weeks of PEG-IFN therapy could represent a sufficient treatment duration for selected patients with HCV genotype 2 and undetectable HCV-RNA at week 4 of treatment.

Since IFN treatment, including PEG-IFN, can cause medical conditions such as depression, interstitial pneumonia, diabetes mellitus, thyroid disease, leukopenia, thrombocytopenia and flu-like symptoms, its duration should preferably be minimal [21]. Therefore, to minimize the possibility of such side effects, and additionally, in consideration of the cost of IFN treatment, reducing the length of treatment is desirable [22,23]. The combination therapy with PEG-IFN and RBV for 24 weeks has been shown to be more effective, and it has become the present standard care [11]. However, in some cases, and especially in older patients, RBV cannot be administered because of possible side effects, including severe anemia. As patients tend to be older in Japan than in Western countries, more therapeutic options are needed.

Recently, there have been several reports about the importance of interleukin-28B (IL28B) SNP in the prediction of SVR in HCV genotype 2-patients treated with PEG-IFN-alfa plus RBV therapy [20,24-28]. IL28B SNP rs8099917 was determined in 11 HCV genotype 2-patients treated with IFN monotherapy, revealing that 5 and 6, respectively, had major (TT) and minor (TG and GG) phenotypes and that 2 null-responders had TG phenotypes, and 4 had relapsed and 5 had SVR [[29], data not shown]. It is possible that IL28B SNP could improve the SVR rate in non-RVR patients and non-responder patients by changing monotherapy to combination therapy [30], but further studies will be needed.

Recently, there have been reports that in HCV genotype 2/3-infected patients with a very rapid viral response (vRVR), i.e. HCV RNA below 1,000 IU/mL on day 7, the combination PEG-IFN plus RBV treatment could be shortened to 12-16 weeks if no dose reduction had been made [31]. Further studies will be needed to reveal how long patients obtaining RVR should be treated. Our study indicates that PEG-IFN monotherapy for 24 weeks or less is sufficient to treat patients with HCV genotype 2, especially those who become negative for HCV RNA by week 4 of treatment. On the other hand, RBV combination therapy should be considered in those whose HCV RNA becomes seropositive by week 4 of treatment. Our results need to be confirmed by a randomized control study as soon as possible. Our previous studies showed that the overall SVR rate of HCV genotype 2-patients treated with PEG-IFN-alfa-2b plus RBV for 16-48 weeks was 82.6% [30], and the SVR rate of combination treatments for some patients infected with HCV genotype 2 was superior to that of PEG-IFN-alfa-2a monotherapy (75.5%) in the present study. Although our retrospective study had a rather small sample size with various treatment durations, our results may provide support to the future design of personalized therapy for HCV genotype 2-patients in certain situations where they cannot be treated with RBV.

## Conclusion

PEG-IFN-alfa-2a monotherapy for 24 weeks or less might be sufficient to treat selected patients with HCV genotype 2, especially those with low viral load and becoming negative for HCV RNA by week 4 of treatment. If we could use IL28B SNP and vRVR for selecting patients, PEG-IFN-alfa-2a monotherapy would be sufficient to allow some HCV genotype 2-patients to clear HCV.

## Competing interests

The authors declare that they have no competing interests.

## Authors' contributions

RE and FI participated in the study design, coordination of statistical analysis and drafted the manuscript. TKanda helped to draft the manuscript. FI, TKurihara, KFukai, KFujiwara, MA, TKanda, RM, YY and OY saw the patients and collected samples. All authors contributed to interpretation of the results, and have read and approved the final manuscript.
